# Enantioseparation Using Cellulose Tris(3,5-dimethylphenylcarbamate) as Chiral Stationary Phase for HPLC: Influence of Molecular Weight of Cellulose †

**DOI:** 10.3390/molecules21111484

**Published:** 2016-11-08

**Authors:** Yuji Okada, Chiyo Yamamoto, Masami Kamigaito, Yuan Gao, Jun Shen, Yoshio Okamoto

**Affiliations:** 1 Graduate School of Engineering, Nagoya University, Furo-cho, Chikusa-ku, Nagoya 464-8603, Japan; column_okd@yahoo.co.jp (Y.O.); chiyo@chem.suzuka-ct.ac.jp (C.Y.); kamigait@apchem.nagoya-u.ac.jp (M.K.); 2 Polymer Materials Research Center, Key Laboratory of Superlight Materials and Surface Technology, Ministry of Education, College of Materials Science and Chemical Engineering, Harbin Engineering University, Harbin 150001, China; gaoyuan1@hrbeu.edu.cn

**Keywords:** chiral separation, enantiomer, phenylcarbamate, polysaccharide

## Abstract

The cellulose oligomers with different degrees of polymerization (DP), 7, 11, 18, 24, 26, 40 and 52, were prepared by hydrolysis of microcrystalline cellulose with phosphoric acid. These oligomers including the starting microcrystalline cellulose (DP 124) were converted to tris(3,5-dimethylphenylcarbamate) (CDMPC) derivatives by the reaction with an excess of 3,5-dimethylphenyl isocyanate to be used as the chiral stationary phase (CSP) in high-performance liquid chromatography (HPLC). The structures of the CDMPC derivatives were investigated by infrared spectroscopy (IR), ^1^H-NMR, circular dichroism (CD) and size exclusion chromatography (SEC), and the DPs of the derivatives estimated by SEC agreed with those estimated by ^1^H-NMR. After coating the derivatives on silica gel, their chiral recognition abilities were evaluated using eight racemates under a normal phase condition with a hexane-2-propanol (99/1) mixture as an eluent. The chiral recognition abilities of 7- and 11-mers, particularly the former, were lower than those of the higher oligomers from DP 18 to 52, which had rather similar abilities to that of 124-mer, although the abilities depended on the racemates. DP 18 seems to be sufficient for CDMPC to exhibit chiral recognition similar to that of the CDMPC with larger DPs.

## 1. Introduction

The enantioseparation by high-performance liquid chromatography (HPLC) using chiral stationary phases (CSPs) is one of the most popular methods for determining the enantiomeric purity of chiral compounds [1,2]. This method is also effective for obtaining enantiomerically pure compounds, and in the past three decades, many CSPs for this separation method have been developed [1,2,3,4,5]. Polysaccharide derivatives, such as 3,5-dimethylphenylcarbamates of cellulose (CDMPC) and amylose, are some of the most popular CSPs [6,7,8], and more than 80% of chiral compounds may be resolvable on these CSPs. The CSPs are usually prepared from the polysaccharides with a degree of polymerization (DP) greater than 100, because the polysaccharide derivatives with lower DPs are more soluble in organic solvents. The high solubility of CSPs is a serious problem due to the restriction in the selection of eluents. On the other hand, enantioseparation abilities of oligomeric amylose with DP 2~7 and oligomeric cellulose with DP 2 and 4 have previously been evaluated as 3,5-dimethylphenylcarbamates [9], and these oligomers’ abilities were very different from those of corresponding polysaccharides. The chiral recognition abilities of phenylcarbamate and 3,5-dichlorophenylcarbamate of cellulose with DP 15 were also evaluated by Kasuya et al. [10]. In the present study, in order to know how the DP of cellulose influences the chiral recognition ability, we prepared celluloses with lower DPs by hydrolysis of microcrystalline cellulose (DP 124) in phosphoric acid according to the method by Isogai et al. [11], and their enantioseparation abilities in HPLC were evaluated as the 3,5-dimethylphenylcarbamates coated the silica gel.

## 2. Results and Discussion 

### 2.1. Structure of CDMPCs

The cellulose oligomers, **Cel**-**1**~**7**, with various DPs (Table 1) were obtained by changing the reaction conditions and the precipitation solvents of the products with the method by Isogai et al. [11]. These oligomers were allowed to react with an excess of 3,5-dimethylphenyl isocyanate to synthesize the tris(3,5-dimethylphenylcarbamate)s (CDMPCs) with various DPs, as shown in Table 2. CDMPC-18 and -24 had different DPs, 18 and 24, although these were obtained from the same **Cel-3**. CDMPC-18 was obtained in a higher yield compared with that of CDMPC-24. Low molecular weight CDMPC seems to still remain in CDMPC-18, leading to a lower DP.

**Figure 1:**
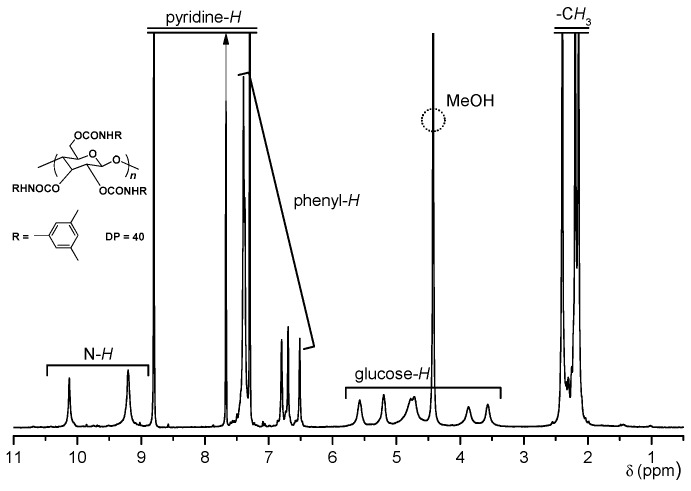
^1^H-NMR spectrum of cellulose tris(3,5-dimethylphenylcarbamate) with DP 40 in pyridine-*d*_5_ at 80 °C (500 MHz).

The ^1^H-NMR spectrum of CDMPC-40 is shown in Figure 1. The peaks were reasonably assigned to the structure of the triscarbamate with the peak intensity ratio of glucose-H, 7.00; -CH_3_, 17.6; phenyl-H, 9.3; N-H, 3.0, although small peaks, which may be assigned to chain end units, were observed.

**Figure 2:**
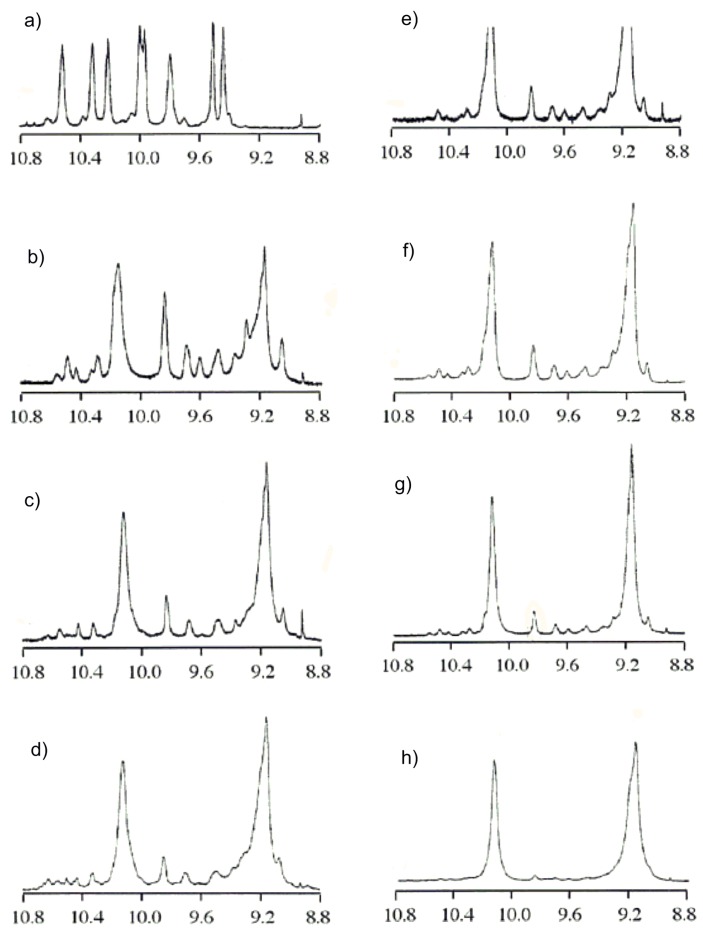
^1^H-NMR spectra of CDMPC-2 (**a**); CDMPC-7 (**b**); CDMPC-11 (**c**); CDMPC-18 (**d**); CDMPC-19 (**e**); CDMPC-24 (**f**); CDMPC-26 (**g**) and CDMPC-124 (**h**) in pyridine-*d*_5_ at 80 °C (400 MHz).

The structures of the other CDMPCs were investigated using the NH protons of carbamate residues including that of cellobiose, and the spectra are shown in Figure 2. The spectrum of the cellobiose derivative is different from those of the higher celluloses. The spectrum of CDMPC-124 with DP 124 is very similar to that of CDMPC-40, showing two intense peaks at 9.20 and 10.15 ppm, which are assigned to the carbamates of the inner glucose residues of cellulose chains. The peak intensities of the two peaks are 1:2, respectively, and the former is assigned to the carbamate at the 6-position and the latter to those at the 2,3-positions on the basis of our previous study on the regioselectively substituted derivatives [12].

Besides these two peaks, many small peaks, which may be assigned to the carbamate groups of the chain end glucose units, can be seen, particularly for low-DP oligomers. Although the detailed assignment of each peak is difficult at this moment, the following estimation is possible. Figure 2b for CDMPC-7 with a DP of 7.3 shows a clear peak at 9.85 ppm. The intensity ratio of this peak to the peak at 10.15 ppm is 2:5. If the peak at 9.85 ppm is ascribed to two protons of the carbamates at the terminal units, the DP of CDMPC-7 is estimated to be 7, which agrees with DP 7.3 obtained by SEC. The relative intensity of the peak at 10.15 to the peak at 9.85 in Figure 2 decreased as the DP increased. The DPs obtained by the NMR method nearly agreed with those obtained by SEC.

The NMR spectra of CDMPC-11 and -18 shown in Figure 2c,d, respectively, are slightly different from those of CDMPC-7 and -26. CDMPC-11 and -18 were synthesized in a mixture of DMA-LiCl-pyridine, whereas the others were in pyridine. The spectra 2c and 2d show no clear peak at 9.6 ppm, while the others show a small peak. The reaction in a DMA-LiCl-pyridine mixture may induce the ring-opening of the reducing glucose end.

**Figure 3:**
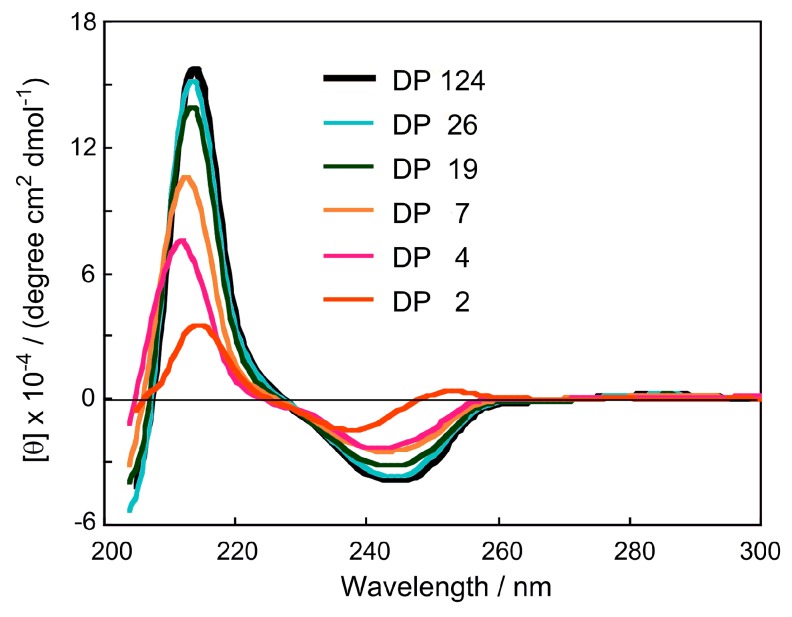
CD spectra of CDMPC-2, 4, 7, 19, 26 and 124 in THF.

The CD spectra of CDMPC-2, CDMPC-4, CDMPC-7, CDMPC-19, CDMPC-26 and CDMPC-124 are shown in Figure 3. The CD intensity was calculated on the basis of a glucose unit. The spectral pattern of the dimer CDMPC-2 is different from the others and shows a weaker intensity compared with higher oligomers. The tetramer CDMPC-4 and other higher oligomers show a similar spectral pattern and their intensities clearly increase with an increase of the DP until DP 26. The intensity difference between DP 26 and 124 is very small, suggesting that in solution, a regular helical structure [13] of a CDMPC chain may be constructed around DP 26. Around this DP value, the influence of chain end units on the CD intensity may be negligible.

### 2.2. Chiral Recognition Ability of CDMPCs in HPLC

**Figure 4:**
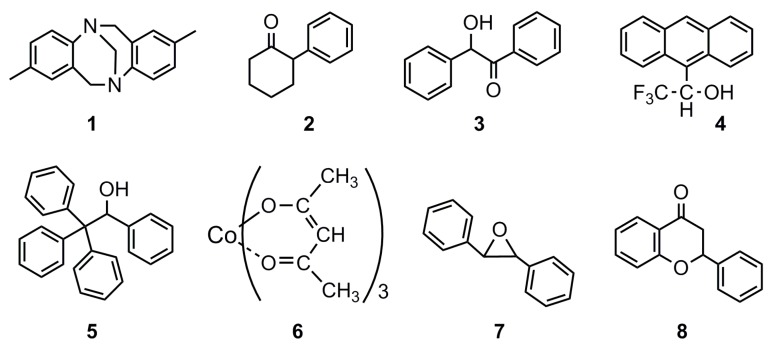
Racemates used for evaluation of CSPs by HPLC.

The chiral recognition of the CDMPCs from DP 2 to 124 was evaluated as the CSPs in HPLC using eight racemates, **1**–**8**, shown in Figure 4. These racemates have often been used in our previous studies to evaluate the chiral recognition ability of polysaccharide-based CSPs [9,14], and many commercially available cellulose- and amylose-based CSPs are well known to show high characteristic separation factors (α values) to these racemates [7,8,9]. The results of the HPLC separation are summarized in Table 3. Since the CDMPC derivatives with low DPs are rather soluble in hexane with higher contents of 2-propanol, we had to use pure hexane or hexane containing 1% or 2% 2-propanol as an eluent, except for the CDMPC with DPs above 40 for which a typical eluent H/I = 90/10 for polysaccharide-based CSPs was able to be used, as shown in the last column of the table. In the table, retention factor (k_1_’) for the first eluted isomer was obtained as (t_1_−t_0_)/t_0_ using the elution times t_0_ and t_1_ for the non-retained compound 1,3,5-tri-tert-butylbenzene [15] and the first eluted isomer, respectively. The separation factors (α) have been obtained as the ratio of k_2_’/k_1_’, where k_2_’ is the retention factor for the second eluted isomer.

CDMPC-2, which cannot have a helical chain structure, exhibited a chiral recognition different from that of CDMPC-4 and -7. For instance, **7** and **8** were efficiently resolved only on CDMPC-2, while **1** and **2** were not resolved only on CDMPC-2. CDMPC-7 efficiently resolved **4**, although CDMPC-2 did not. The chiral recognition abilities of CDMPC-7, -11 and -18 appear to increase with an increase of the DP, and the CDMPC derivatives with a DP greater than 18 seem to have a similar chiral recognition ability. These results seem to agree with the results reported by Kasuya et al. on the cellulose trisphenylcarbamate with DP 15 [10], which showed a slightly lower chiral recognition compared to the same derivative with a much higher DP. The (+)-enantiomer of **7** was eluted first on CDMPC-2 and -11, while the (–)-enantiomer was eluted on CDMPC-7. The reason for this reversed enantioselectivity on these three CSPs is not clear at this moment. The separation results on CDMPC-124 with H/I = 90/10 are rather similar to those on the CDMPC obtained with the microcrystalline cellulose Avicel, which probably has a DP of about 200 [14]. These results indicate that DP 18 is sufficient for CDMPC to show chiral recognition similar to the CDMPC with larger DPs. This DP value of 18 is slightly smaller than the DP 26 that was necessary for the oligomers to reach a constant CD intensity, as shown in Figure 2. This discrepancy seems reasonable because the CD spectra were measured in a solution, while the HPLC analysis was performed in a hexane/2-propanol (99/1) mixture where the oligomers are totally insoluble. Since, in the solution, chain end groups are more dynamic, a longer chain must be required in order to neglect this effect.

The influence of an eluent, H/I = 90/10, was examined on CDMPC-124. The capacity factors k_1_’ for the all racemates became smaller with this eluent. This more polar eluent must weaken the hydrogen bond interaction between the CSP and the racemates, resulting in the decrease of the k_1_’. However, the influence of the eluent on the chiral recognition depended on the racemates, and the α value increased for **1** and **5**, whereas it decreased for **3**, **4**, **6** and **7**. The influence of 2-propanol content in the eluent on the enantioselective interaction between the CSP and racemates is not simply explained.

### 2.3. Chiral Recognition Ability of CDMPC in NMR

**Figure 5:**
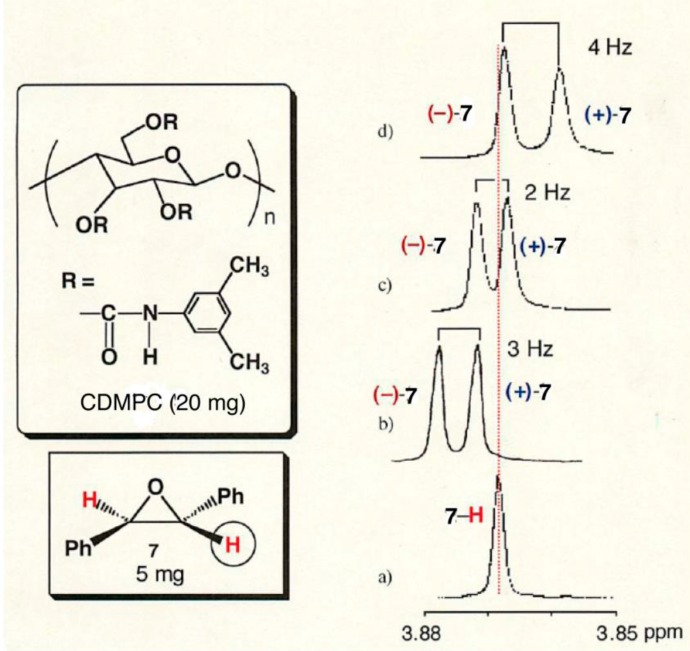
^1^H-NMR of **7** in the absence (**a**) and presence of CDMPC-4 (**b**); CDMPC-7 (**c**) and CDMPC-26 (**d**) (500 MHz, 23 °C, chloroform-*d*_1_, [CDMPC]/[**7**] = 1.3).

The CDMPCs with DPs below 26 are soluble in chloroform, which enabled us to investigate their chiral recognition abilities by ^1^H-NMR. Figure 5 shows the NMR spectra of the oxirane proton of racemic **7** in the absence (a) and presence (b, c and d) of CDMPC-4, -7 and -26, respectively, in chloroform-*d*_1_. The free oxirane proton is observed as a single peak at 3.868 ppm in the absence of CDMPC, while in the presence of 1.3-times excess CDMPC based on glucose units, the peak is split into two peaks of enantiomers showing (−)-**7** down-field to (+)-**7**. In the presence of CDMPC-4, both enantiomers move down-field compared to the free **7**, indicating that (−)-**7** interacts more tightly with CDMPC-4. On the other hand, in the presence of CDMPC-26, both enantiomers move up-field to the free **7**, indicating that (+)-**7** interacts more tightly with CDMPC-26. In addition, in the presence of CDMPC-7, (−)-**7** moves down-field and (+)-**7** up-field. These results are well correlated with the HPLC results shown in Table 2, where on the CDMPC-4–based CSP, (−)-**7** eluted as the second peak, and on the CDMPC-26–based CSP, (+)-**7** eluted as the second peak. The CSP CDMPC-7 showed a lower chiral recognition to **7**.

**Figure 6:**
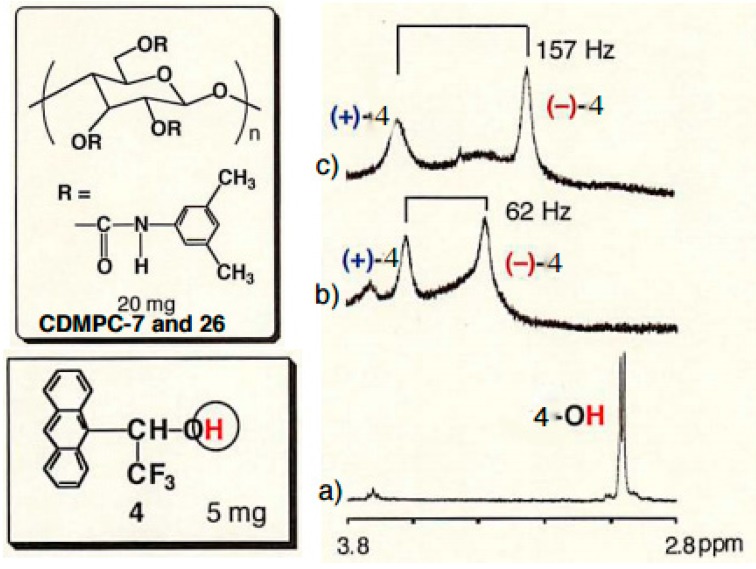
^1^H-NMR spectra of free **4** (**a**) and in the presence of CDMPC-7 (**b**) and CDMPC-26 (**c**) (400 MHz, 23 °C, chloroform-*d*_1_).

Figure 6 shows the ^1^H-NMR of the hydroxy group of **4** in the absence (a) and presence of CDMPC-7 (b) and -26 (c) in chloroform-*d*_1_. The OH proton of (+)-**4** is more down-shifted than (–)-**4** by CDMPC-7 and -26. This result agreed with that of the HPLC separation of **4** on both CDMPCs, where (+)-**4** was more retained as the second peak, giving the separation factors 2.05 and 2.21, respectively. The enantiomers of benzoin **3** and 2,2′-dihydroxy-1,1′-binaphthyl were also recognized by ^1^H-NMR. However, the enantiomers of racemates **1**, **2**, **5** and **8** showed no separate peaks due to their enantiomers.

## 3. Materials and Methods

### 3.1. Chemicals

Microcrystalline cellulose (Avicel) was obtained from Merck Chemicals GmbH (Darmstadt, Germany). 3,5-Dimethylphenyl isocyanate was kindly supplied by Daicel Co. (Osaka, Japan). The solvents, *N*,*N*-dimethylacetamide (DMA), pyridine, hexane, 2-propanol and chloroform were obtained from Kanto Chemicals (Tokyo, Japan). Phosphoric acid was purchased from Hayashi Pure Chemical (Osaka, Japan). Wide-pore silica gel (Daiso gel SP-1000) with a mean particle size 7 μm and a mean pore diameter 100 nm was kindly supplied by Daiso Chemicals (current name: Osaka Soda (Osaka, Japan)). Racemates were commercially available or synthesized by the usual methods.

### 3.2. Measurements

The ^1^H-NMR spectra of the products were measured with a Varian Gemini-2000 (400 MHz) or a Bruker (500 MHz) spectrometer (Bruker, Billerica, MA, USA) in pyridine-*d*_5_ at 80 °C. IR spectra were obtained with a JASCO FT/IR-620 spectrophotometer (JASCO, Tokyo, Japan). The CD spectra of 3,5-dimethylphenylcarbamates of cellulose were measured on a JASCO J-720 spectrophotometer using a quartz cell with a path length of 0.10 mm. The concentrations of samples in THF were 3.3–4.6 mg/5 mL. Optical rotation was measured with a JASCO P-1030 polarimeter. Thermal analysis of products was performed on a SEIKO SSC/5200TG instrument. The molecular weights (Mn) and its distribution (Mw/Mn) or DPs of the celluloses were estimated as the tris(3,5-dimethylphenylcarbamate)s by SEC on Shodex GPC system-21 at 40 °C using polystyrene standards and tetrahydrofuran (THF) as solvent. Two SEC columns, Shodex KF-806 and KF-803, were used in a series. For the samples with DP 40 and 52, the SEC on Waters 600 GPC system was used with three SEC columns, Styragel HR 0.5, HR 3 and HR 4. The HPLC analysis of racemates was carried out with a JASCO HPLC system equipped with a PU-980 pump and a JASCO OR-990 polarimetric detector or a JASCO CD-2095 detector.

### 3.3. Preparation of Cellulose with Lower DPs

Seven cellulose oligomers with lower DPs, **Cel-1**~**7**, were prepared according to the method by Isogai, et al. [11]. As an example, the preparation of **Cel-1** is presented. Microcrystalline cellulose (Avicel, 10 g) was added to a mixture of 85% phosphoric acid (187 mL) and water (7.3 mL), and left without stirring for eight weeks at room temperature to dissolve the cellulose. The obtained solution was added into a large excess of water and insoluble part (**Cel-1**) was separated by filtration; yield 0.77 g (7.7%). The results of preparation are summarized in Table 1.

### 3.4. Synthesis of 3,5-Dimethylphenylcarbamates of Cellulose with Various DPs

Reaction of the microcrystalline cellulose or its oligomers with 3,5-dimethylphenyl isocyanate was carried out in a two-necked flask under nitrogen atmosphere. The cellulose was first placed in a flask and dried under vacuum for several hours, and then dry pyridine or a mixture of DMA, lithium chloride and dry pyridine (DMA-Li-py) was added to completely dissolve the cellulose. The mixture heated to 80 °C and an excess 3,5-dimethylphenyl isocyanate (1.3~1.5 equivalent to hydroxyl groups) was added under stirring. After reacted for 20 h, the reaction mixture was added into a large excess of methanol or a methanol-water mixture to precipitate the produced 3,5-dimethylphenylcabamate derivatives. The obtained derivatives are summarized in Table 2. The DP value of each oligomer was obtained by dividing the number-average molecular weight (Mn) with the weight value (603) of one glucose unit. The molecular weight distribution (Mw/Mn) of each derivative was rather narrow except for CDMPC-124, indicating that most derivatives were well fractionated.

The CDMPCs with DP 2 and 4 were synthesized from cellobiose and cellotetraose, respectively, in our previous study [9].

### 3.5. Preparation of CSPs Based on CDMPCs

Macroporous silica gel (Daisogel-7-1000) with particle size 7 μm and pore size 100 nm was treated with 3-aminopropyltriethoxysilane in the presence of pyridine in toluene at 80 °C. To prepare the CSPs based on the CDMPCs, the CDMPCs were coated on the 3-aminopropylsilanized silica gel at the weight ratio of CDMPC to silica gel equal to 20:80 according to our previous method [14]. The obtained CSPs were packed in HPLC tubes (length 25 cm, inner diameter 0.46 or 0.20 cm) by a slurry method.

## 4. Conclusions

The chiral recognition abilities of cellulose oligomers with DP 7, 11, 18, 24, 26, 40 and 52 were evaluated by HPLC as 3,5-dimethylphenylcarbamate derivatives. The derivatives with DP 7 and 11 exhibited rather low chiral recognition, while the oligomers with DPs over 18 showed abilities to similar to that of the derivative of microcrystalline cellulose with DP 124.

## Figures and Tables

**Table 1:** Preparation of cellulose oligomers with lower DPs using Avicel ^a^.

Cellulose Oligomer	Avicel (g)	85% H_3_PO_4_ +H_2_O (mL)	Time (Day)	Stirring	H_2_O ^b^ (L)	Yield g (%)	DP ^c^
**Cel-1**	10	187 + 7.3	56	without	large excess	0.77 (7.7)	7.3
**Cel-2** ^d^	20	347 + 14.6	12	with	4.5	4.91 (25)	11.0
**Cel-3**	10	187 + 7.3	14	without	0.6	8.14 (81)	18.0
**Cel-4**	3	56 + 2	14	without	0.7	1.47 (49)	19.1
**Cel-5**	5	88.5 + 3.7	14	without	1.4	3.85 (77)	23.6
**Cel-6**	1	18.7 + 0.7	3	without	0.25	0.89 (89)	40.2
**Cel-7**	1	18.7 + 2.6	0.7	with	0.25	0.85 (85)	51.6

^a^ Reaction was carried out at room temperature. ^b^ Amount of water used to precipitate cellulose oligomers. ^c^ DP estimated by SEC as tris(3,5-dimethylphenylcarbamate)s. ^d^ Reaction was carried out at 30 °C.

**Table 2:** Preparation of CDMPC derivatives with the DPs from 2 to 124.

CDMPC-	Cellulose Oligomer	Reaction Solvent ^a^	Reaction Time (h)	Solvent for CDMPC ppt ^b^	Yield (%)	Mn × 10^−3^ (Mw/Mn)	DP SEC	DP NMR
2 ^c^	Cellobiose	pyridine		MeOH–H_2_O (4:1)				
4 ^c^	Cellotetraose	pyridine		MeOH–H_2_O (4:1)				
7	**Cel-1**	pyridine	20	MeOH–H_2_O (4:1)	30	4.4 (1.13)	7.3	7
11	**Cel-2**	DMA-Li-py	20	MeOH	55	6.7 (1.36)	11.0	
18	**Cel-3**	DMA-Li-py	24	MeOH–H_2_O (9:1)	99	10.8 (1.71)	18.0	
19	**Cel-4**	pyridine	41	MeOH	18	11.5 (1.36)	19.1	
24	**Cel-3**	pyridine	48	MeOH–H_2_O (9:1)	89	14.2 (1.86)	23.6	19
26	**Cel-5**	pyridine	20	MeOH	55	15.5 (1.40)	25.7	29
40	**Cel-6**	pyridine	72	MeOH	89	24.2 (1.60)	40.2	
52	**Cel-7**	pyridine	17 ^d^	MeOH	85	31.1 (1.65)	51.6	
124	Avicel	pyridine	20	MeOH	82	74.7 (2.90)	124	

^a^ Solvents used for the synthesis of CDMPC derivatives. ^b^ Solvents used for precipitating CDMPC derivatives. ^c^ Data cited from ref. [9]. ^d^ The hydrolysis reaction of cellulose was carried out using 80% phosphoric acid.

**Table 3:** HPLC separation of racemates **1**–**8** on the CSPs prepared from 10 CDMPCs shown in Table 2 ^a^.

CSP=	CDMPC-2 ^b^		CDMPC-4 ^b^		CDMPC-7		CDMPC-11		CDMPC-18		CDMPC-24		CDMPC-26		CDMPC-40 ^d^		CDMPC-52 ^d^		CDMPC-124		CDMPC-124	
Eluent ^c^=	H/I = 98/2		H = 100		H/I = 99/1		H/I = 99/1		H/I = 99/1		H/I = 99/1		H/I = 99/1		H/I = 99/1		H/I = 99/1		H/I = 99/1		H/I = 90/10	
Racemate	k_1_′	α	k_1_′	α	k_1_′	α	k_1_′	α	k_1_′	α	k_1_′	α	k_1_′	α	k_1_′	α	k_1_′	α	k_1_′	α	k_1_′	α
**1**	0.26 (–)	~1	1.54 (+)	1.21	1.33 (+)	1.08	1.44 (+)	1.33	1.49 (+)	1.38	2.19 (+)	1.27	1.81 (+)	1.27	2.21 (+)	1.29	2.49 (+)	1.31	1.85 (+)	1.30	0.74 (+)	1.50
**2**	0.25 (–)	~1	2.13 (–)	1.13	1.50 (–)	1.15	2.31 (–)	1.29	1.57 (–)	1.40	2.56 (–)	1.17	2.14 (–)	1.22	2.47 (–)	1.18	3.03 (–)	1.20	2.33 (–)	1.22	0.91 (–)	1.29
**3**					5.25 (+)	1.16	8.22 (+)	1.16	5.29 (+)	1.42	7.28 (+)	1.43	7.53 (+)	1.42	1.37 (+)	1.67	8.24 (+)	1.57	6.82 (+)	1.54	2.06 (+)	1.40
**4**	1.72	1.00			5.48 (–)	2.05	11.9 (–)	1.71	4.66 (–)	2.94	26.9 (–)	2.41	15.9 (–)	2.21	33.2 (–)	2.34	36.6 (–)	3.04	22.4 (–)	3.19	1.54 (–)	2.60
**5**					3.11 (+)	1.15			4.03 (+)	1.17	6.67 (+)	1.06	5.14 (+)	1.09					5.82 (+)	1.10	1.23 (+)	1.23
**6**					2.58	1.00	2.33	1.00	1.31 (+)	~1	2.00 (+)	1.07	2.18 (+)	~1	2.15 (–)	1.12	1.72 (–)	1.13	1.26 (+)	1.14	0.34 (+)	1.00
**7**	1.38 (+)	1.37	9.38	1.00	0.64 (–)	1.13	0.83 (+)	1.17	0.91 (–)	2.32	1.25 (–)	2.55	1.05 (–)	2.21	1.14 (–)	2.38	1.53 (–)	2.75	1.21 (–)	2.82	0.60 (–)	1.95
**8**	0.29 (+)	1.38	3.70 (+)	~1	2.22 (–)	~1	3.17	1.00	1.23 (–)	1.23	3.42 (–)	1.25	2.86 (–)	1.25	3.21 (–)	1.23	4.00 (–)	1.30	3.00 (–)	1.36	1.14 (–)	1.32

^a^ The sign in the parenthesis is the optical activity of the first eluted isomer. ^b^ Data cited from Reference [9]. ^c^ Eluent: H, hexane; I, 2-propanol. ^d^ The sign in the parenthesis is that of the first eluted isomer for CD detector.
